# Supplementary figures and images for: Bayesian network analysis incorporating genetic anchors complements conventional Mendelian randomization approaches for exploratory analysis of causal relationships in complex data
Source: PLoS Genet. 2020 Mar 2;16(3):e1008198. doi: 10.1371/journal.pgen.1008198 (PMC7067488; doi:10.1371/journal.pgen.1008198)

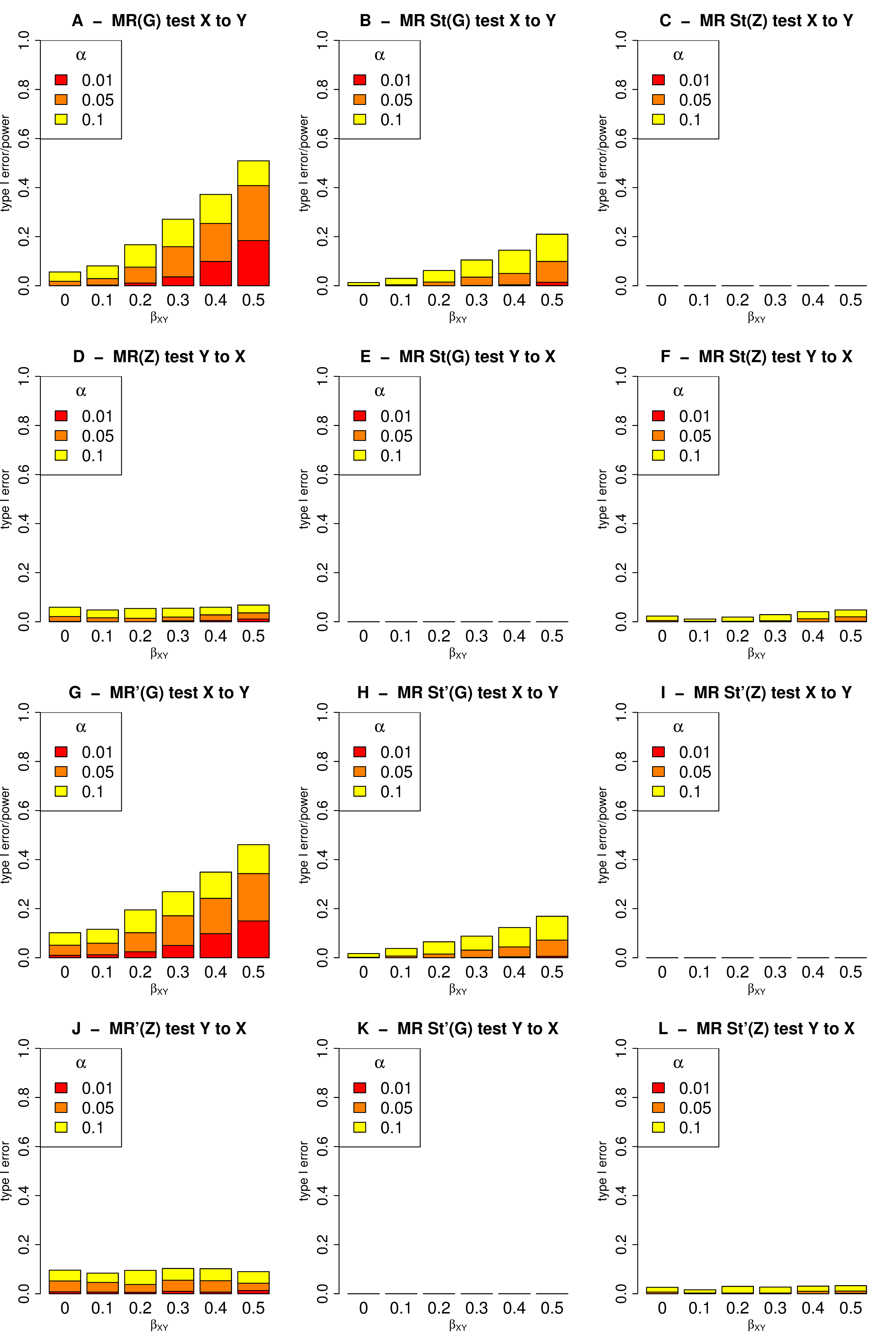

Supplement: S1 Fig — MR and St denote MR and MR Steiger respectively, performed using instrumental variable regression which takes into account the uncertainty of the predicted values in the first-stage regression to calculate the MR p-values. MR’ and St’ denote MR and MR Steiger respectively, performed using two-stage least squares regression without accounting for the uncertainty of the predicted values in the first-stage regression. (TIF) [file pgen.1008198.s001.tif]

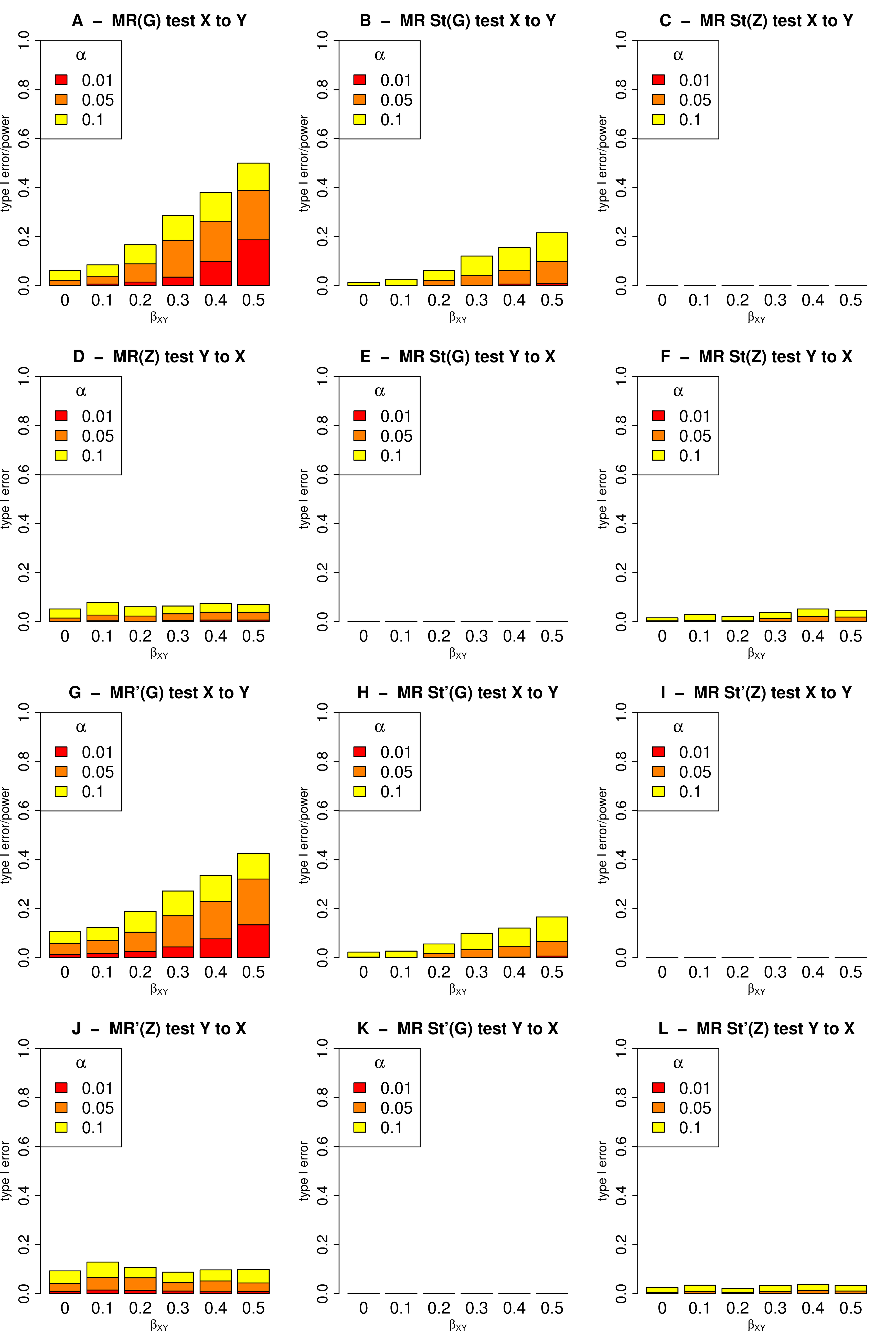

Supplement: S2 Fig — MR and St denote MR and MR Steiger respectively, performed using instrumental variable regression which takes into account the uncertainty of the predicted values in the first-stage regression to calculate the MR p-values. MR’ and St’ denote MR and MR Steiger respectively, performed using two-stage least squares regression without accounting for the uncertainty of the predicted values in the first-stage regression. (TIF) [file pgen.1008198.s002.tif]

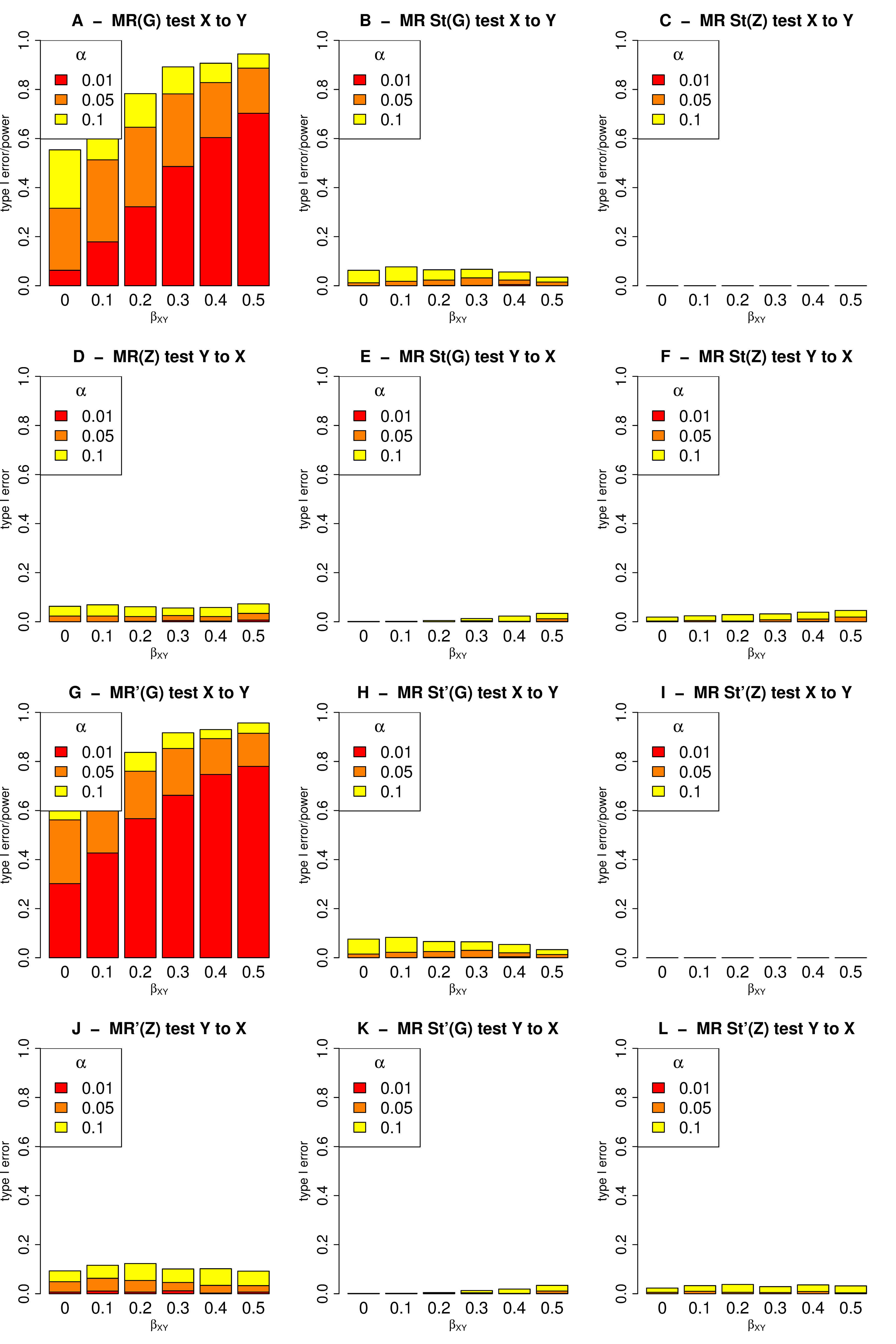

Supplement: S3 Fig — MR and St denote MR and MR Steiger respectively, performed using instrumental variable regression which takes into account the uncertainty of the predicted values in the first-stage regression to calculate the MR p-values. MR’ and St’ denote MR and MR Steiger respectively, performed using two-stage least squares regression without accounting for the uncertainty of the predicted values in the first-stage regression. (TIF) [file pgen.1008198.s003.tif]

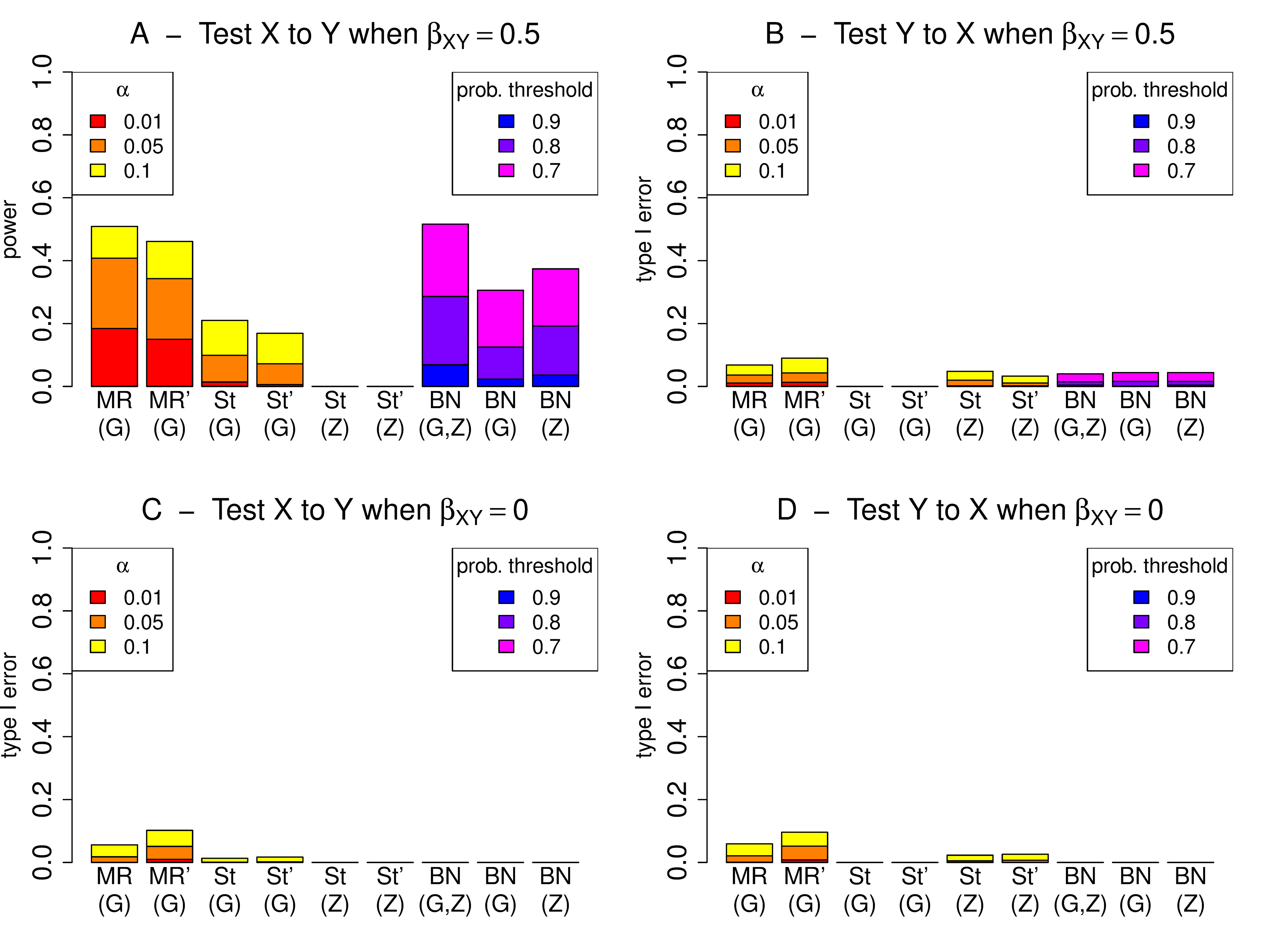

Supplement: S4 Fig — MR and St denote MR and MR Steiger respectively, performed using instrumental variable regression which takes into account the uncertainty of the predicted values in the first-stage regression to calculate the MR p-values. MR’ and St’ denote MR and MR Steiger respectively, performed using two-stage least squares regression without accounting for the uncertainty of the predicted values in the first-stage regression. (TIF) [file pgen.1008198.s004.tif]

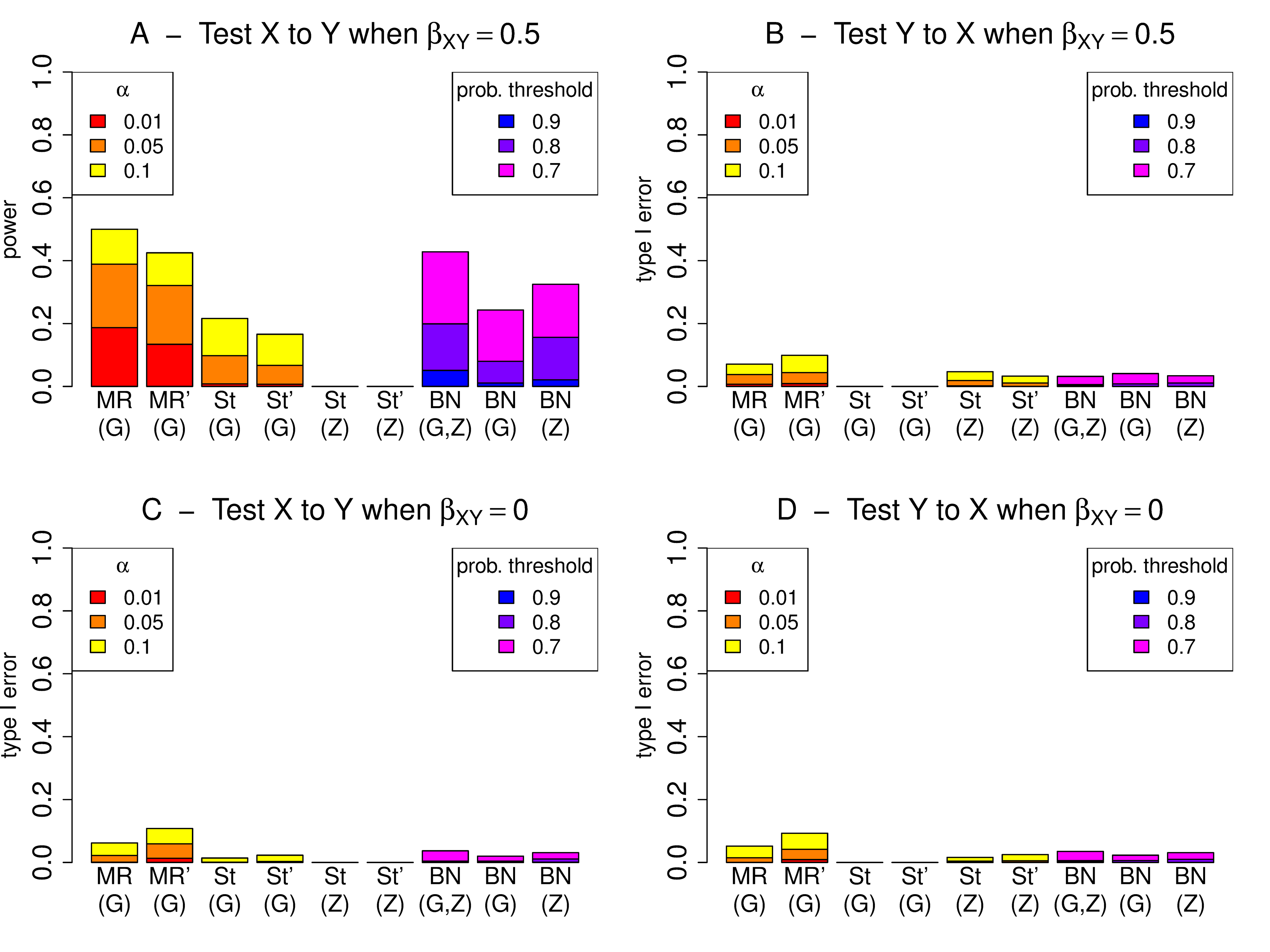

Supplement: S5 Fig — MR and St denote MR and MR Steiger respectively, performed using instrumental variable regression which takes into account the uncertainty of the predicted values in the first-stage regression to calculate the MR p-values. MR’ and St’ denote MR and MR Steiger respectively, performed using two-stage least squares regression without accounting for the uncertainty of the predicted values in the first-stage regression. (TIF) [file pgen.1008198.s005.tif]

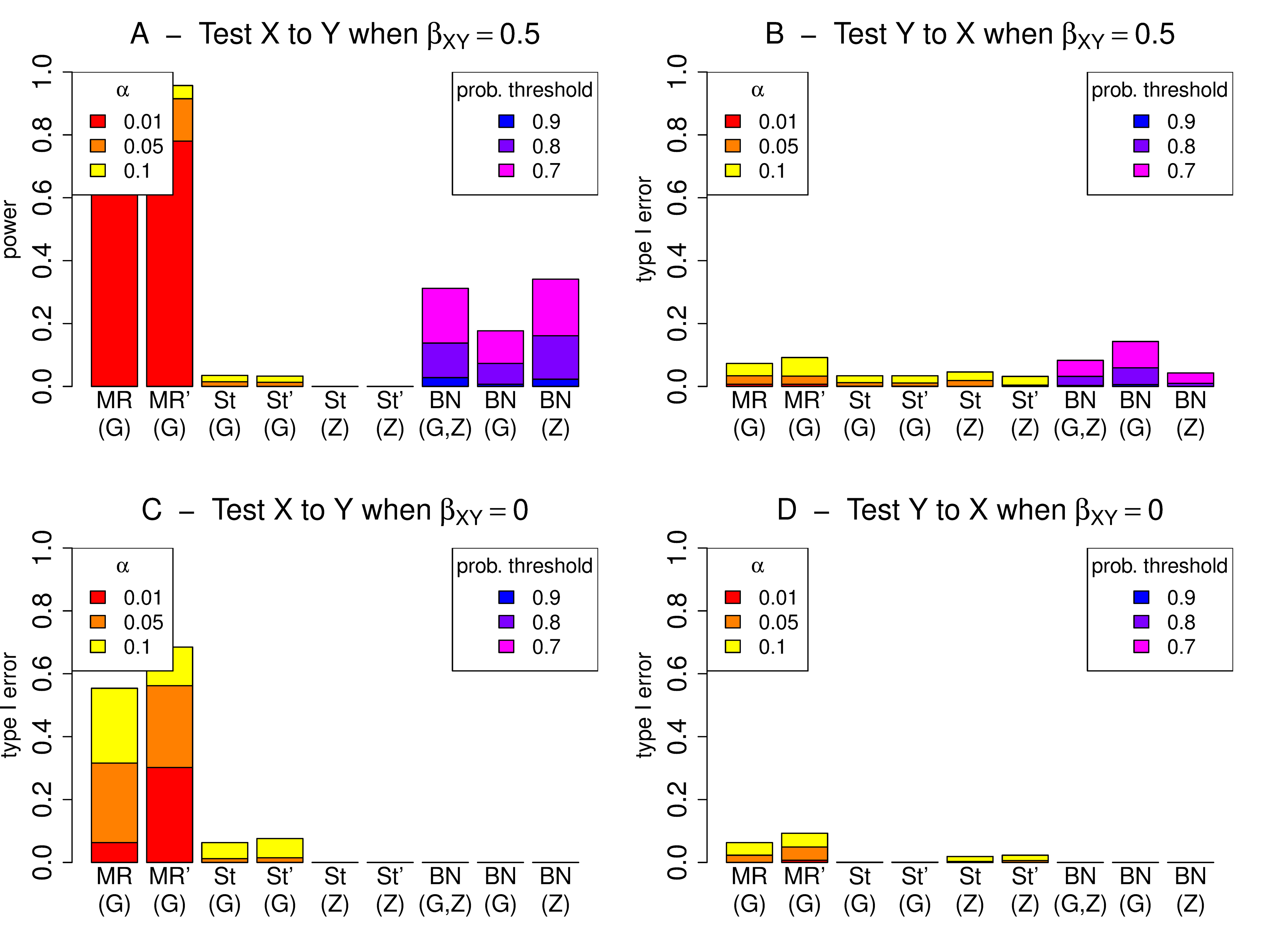

Supplement: S6 Fig — MR and St denote MR and MR Steiger respectively, performed using instrumental variable regression which takes into account the uncertainty of the predicted values in the first-stage regression to calculate the MR p-values. MR’ and St’ denote MR and MR Steiger respectively, performed using two-stage least squares regression without accounting for the uncertainty of the predicted values in the first-stage regression. (TIF) [file pgen.1008198.s006.tif]

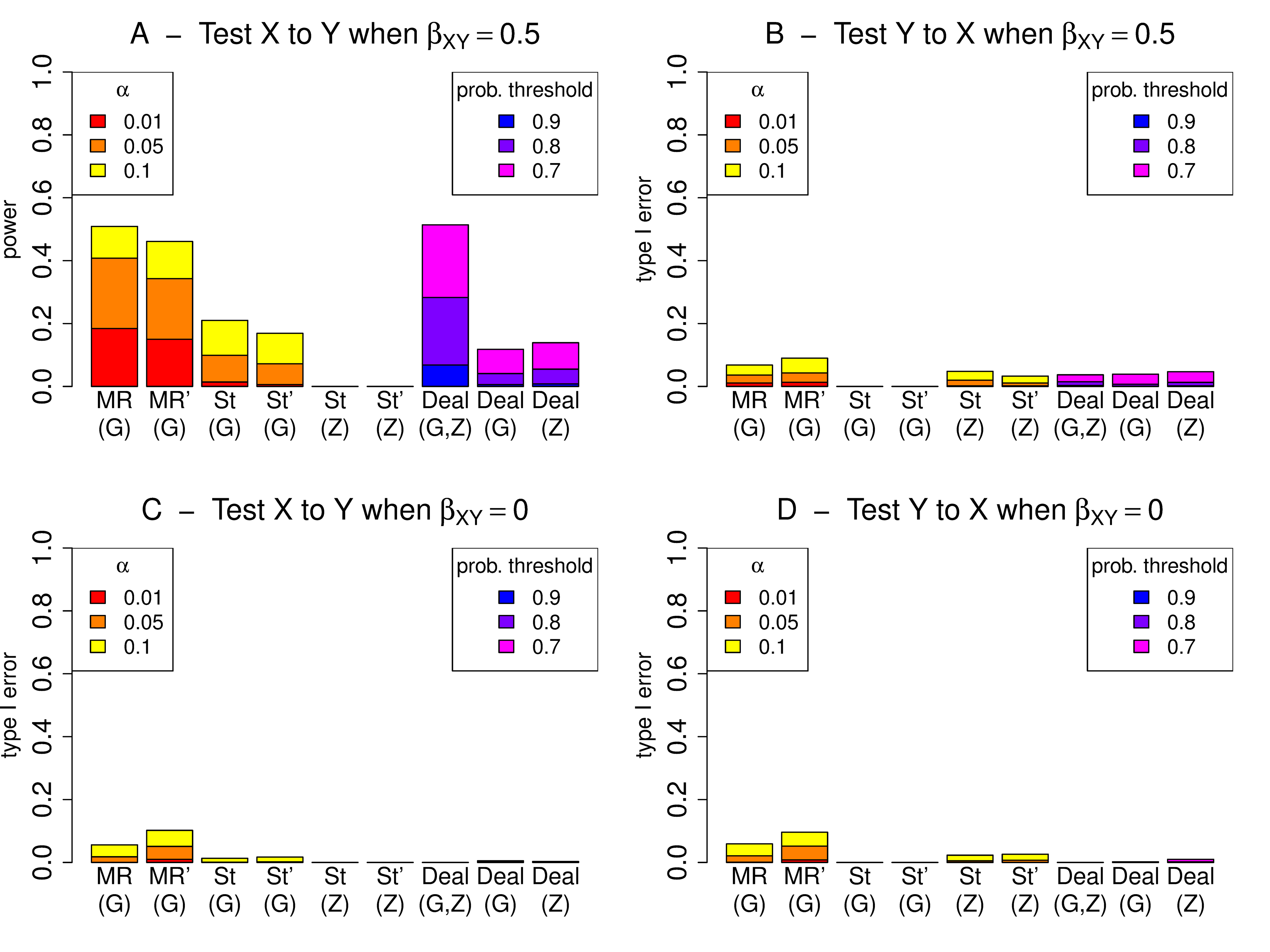

Supplement: S7 Fig — MR and St denote MR and MR Steiger respectively, performed using instrumental variable regression which takes into account the uncertainty of the predicted values in the first-stage regression to calculate the MR p-values. MR’ and St’ denote MR and MR Steiger respectively, performed using two-stage least squares regression without accounting for the uncertainty of the predicted values in the first-stage regression. (TIF) [file pgen.1008198.s007.tif]

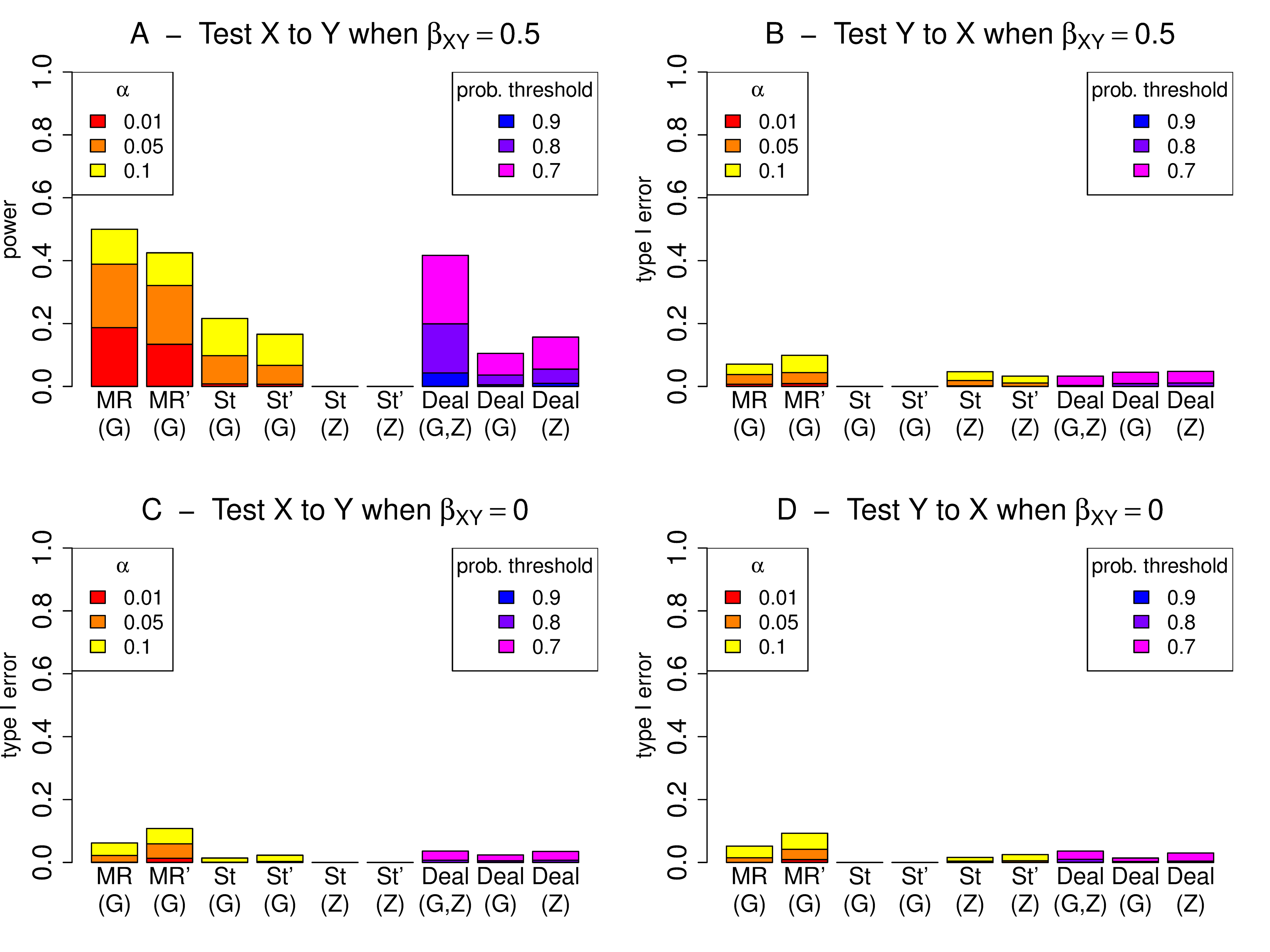

Supplement: S8 Fig — MR and St denote MR and MR Steiger respectively, performed using instrumental variable regression which takes into account the uncertainty of the predicted values in the first-stage regression to calculate the MR p-values. MR’ and St’ denote MR and MR Steiger respectively, performed using two-stage least squares regression without accounting for the uncertainty of the predicted values in the first-stage regression. (TIF) [file pgen.1008198.s008.tif]

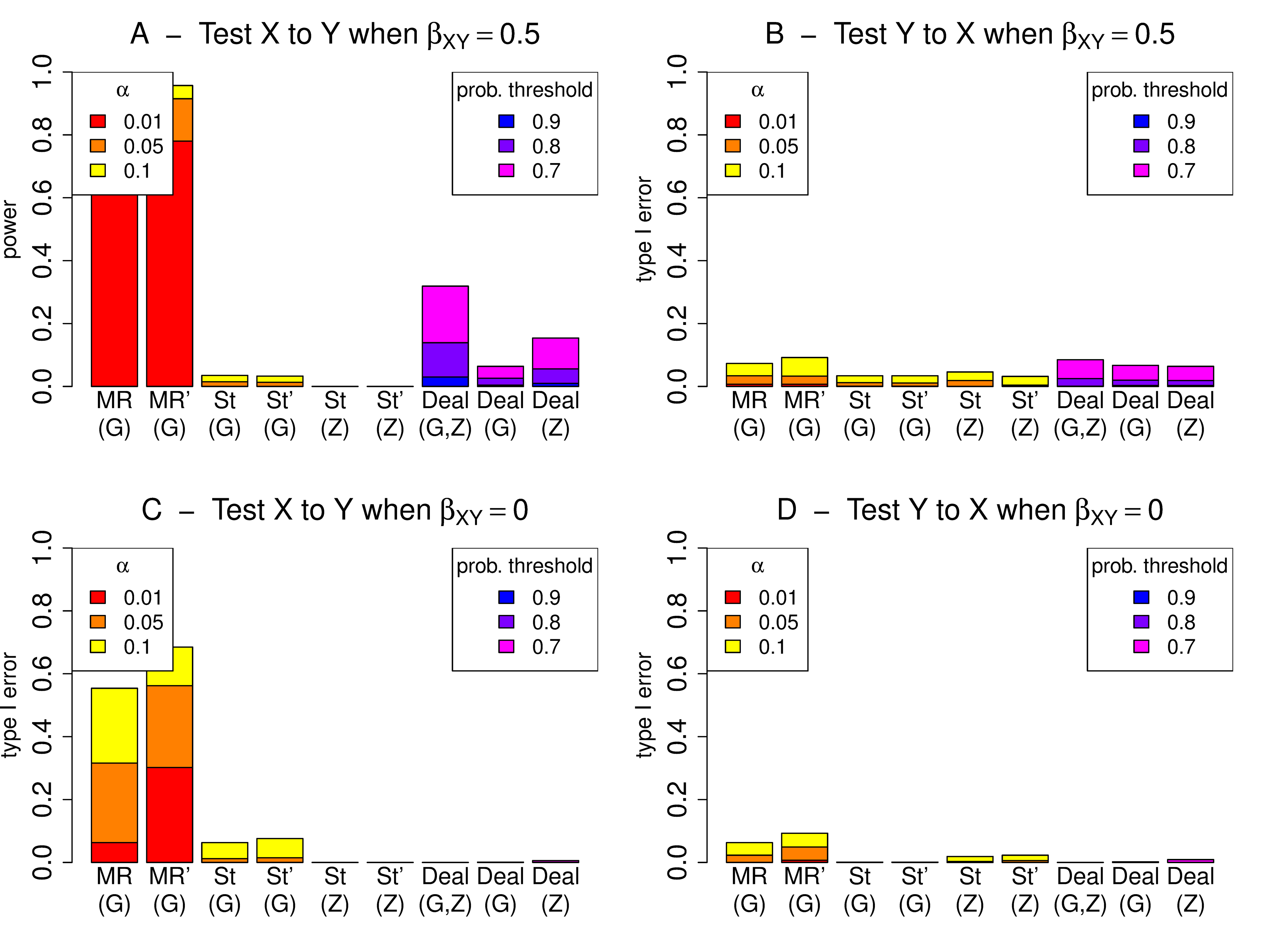

Supplement: S9 Fig — MR and St denote MR and MR Steiger respectively, performed using instrumental variable regression which takes into account the uncertainty of the predicted values in the first-stage regression to calculate the MR p-values. MR’ and St’ denote MR and MR Steiger respectively, performed using two-stage least squares regression without accounting for the uncertainty of the predicted values in the first-stage regression. (TIF) [file pgen.1008198.s009.tif]

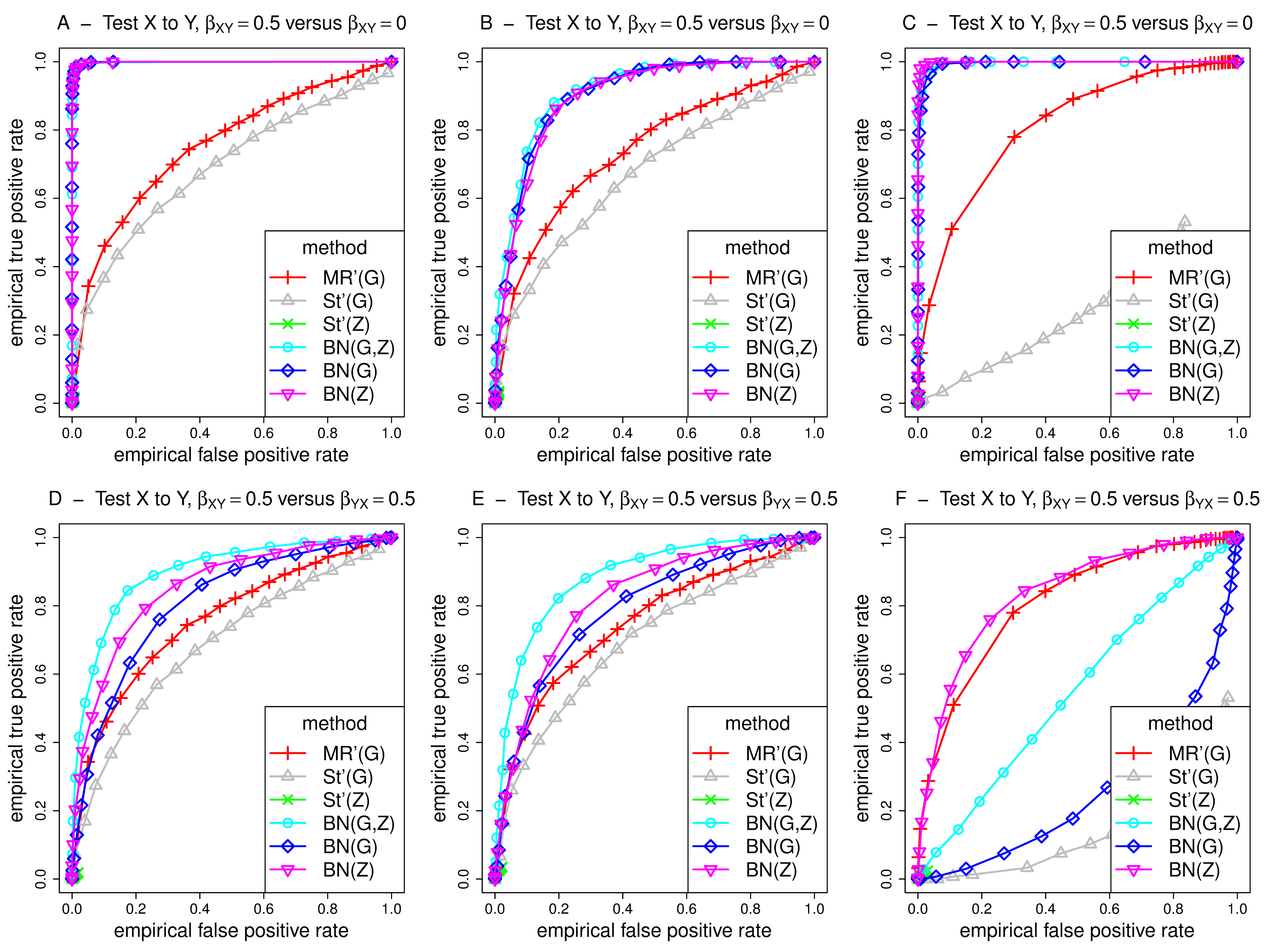

Supplement: S10 Fig — MR’ and St’ denote MR and MR Steiger respectively, performed two-stage least squares regression without accounting for the uncertainty of the predicted values in the first-stage regression. Left hand plots (A, D) are generated under model 1 (no confounding), middle plots (B, E) are generated under model 2 (non-genetic confounding), and right hand plots (C, F) are generated under model 3 (genetic confounding). For the top plots (panels A-C), false positives on the x-axis are counted using simulations when there is no effect (βXY = 0), while for the bottom plots (panels D-F), the false positive rate is calculated by simulating from a model where there is a causal effect from Y to X. (TIF) [file pgen.1008198.s010.tif]

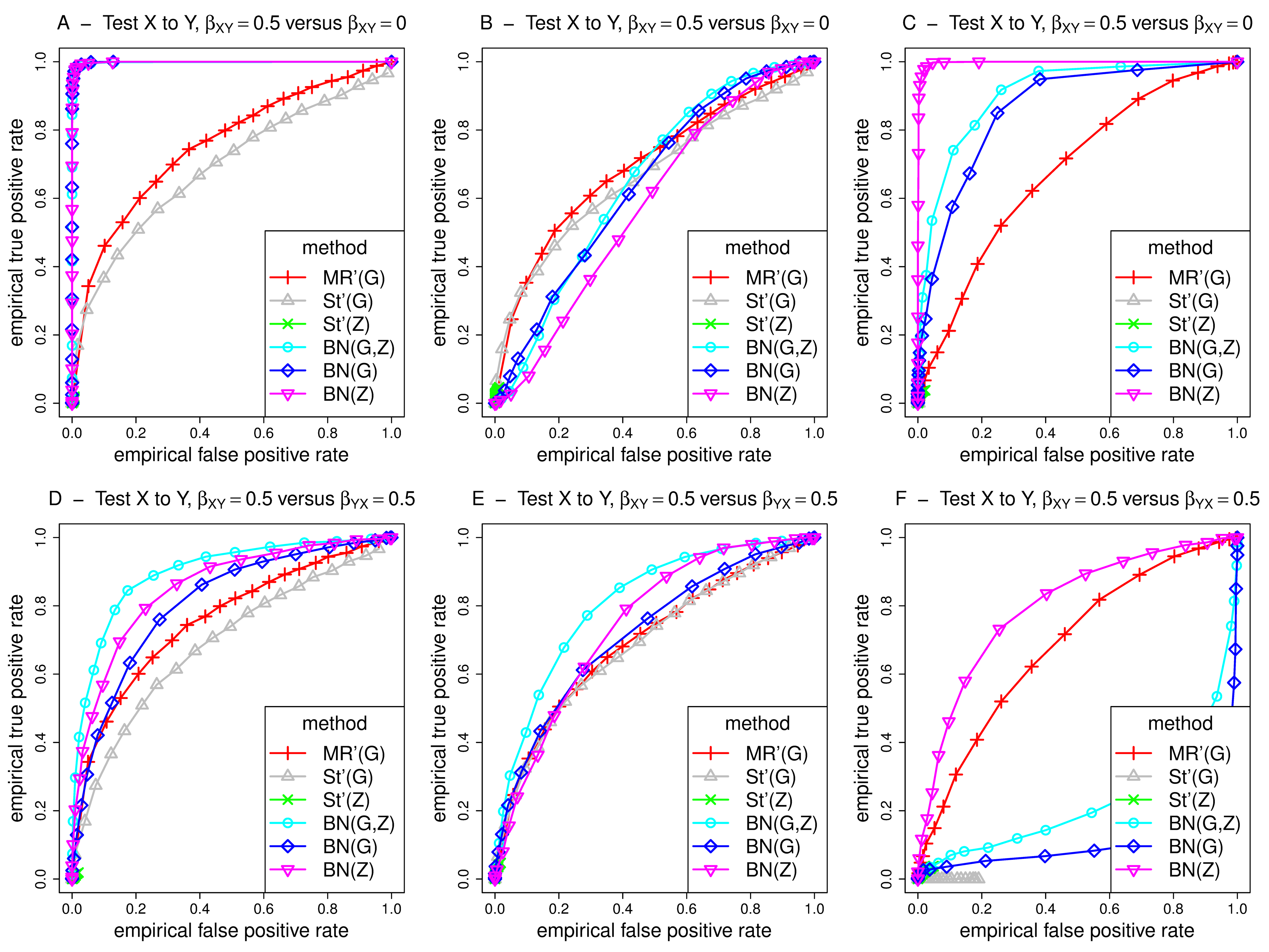

Supplement: S11 Fig — MR’ and St’ denote MR and MR Steiger respectively, performed two-stage least squares regression without accounting for the uncertainty of the predicted values in the first-stage regression. Left hand plots (A, D) are generated under model 1 (no confounding), middle plots (B, E) are generated under model 2 (non-genetic confounding), and right hand plots (C, F) are generated under model 3 (genetic confounding). For the top plots (panels A-C), false positives on the x-axis are counted using simulations when there is no effect (βXY = 0), while for the bottom plots (panels D-F), the false positive rate is calculated by simulating from a model where there is a causal effect from Y to X. (TIF) [file pgen.1008198.s011.tif]

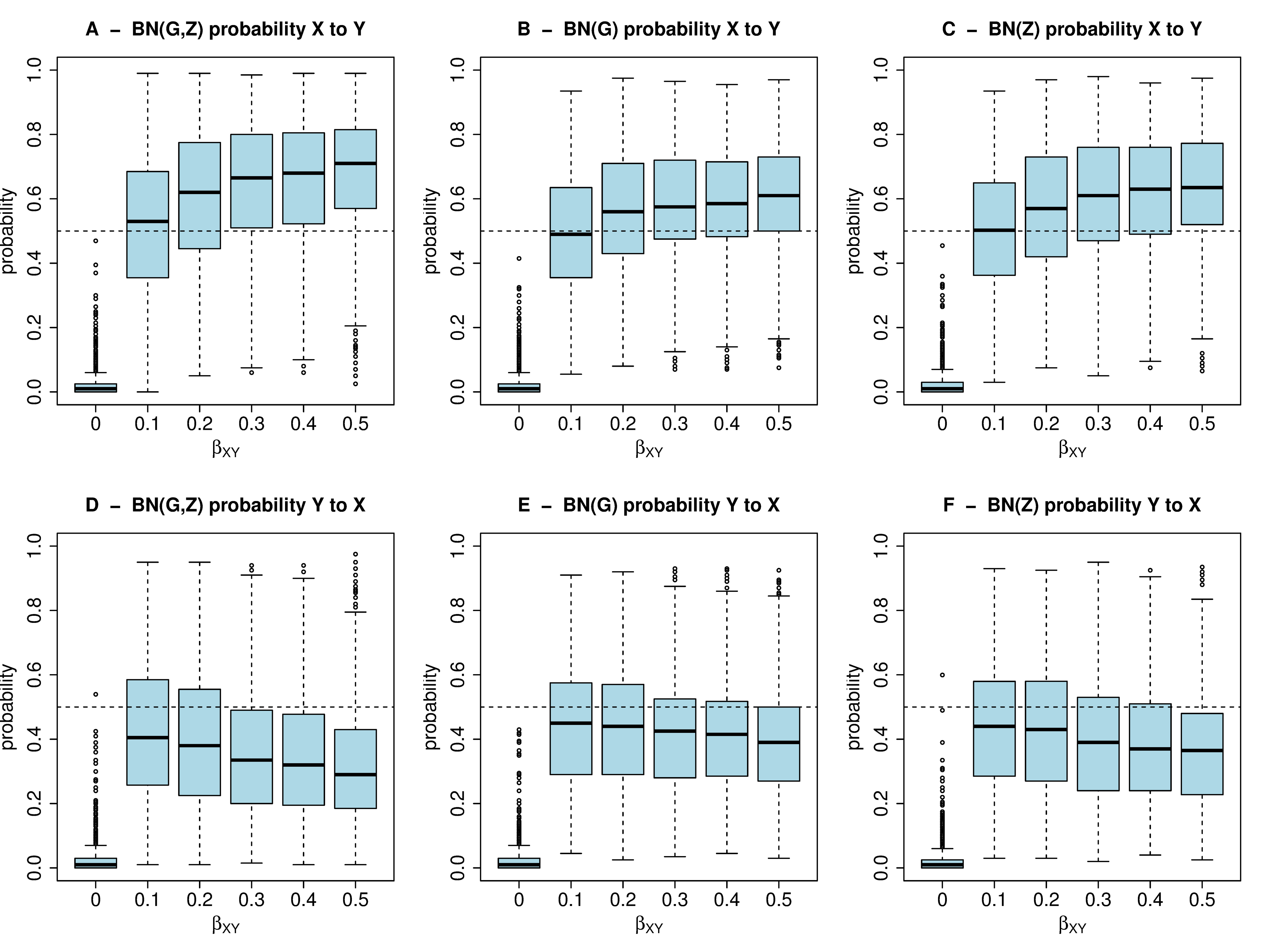

Supplement: S12 Fig — (TIF) [file pgen.1008198.s012.tif]

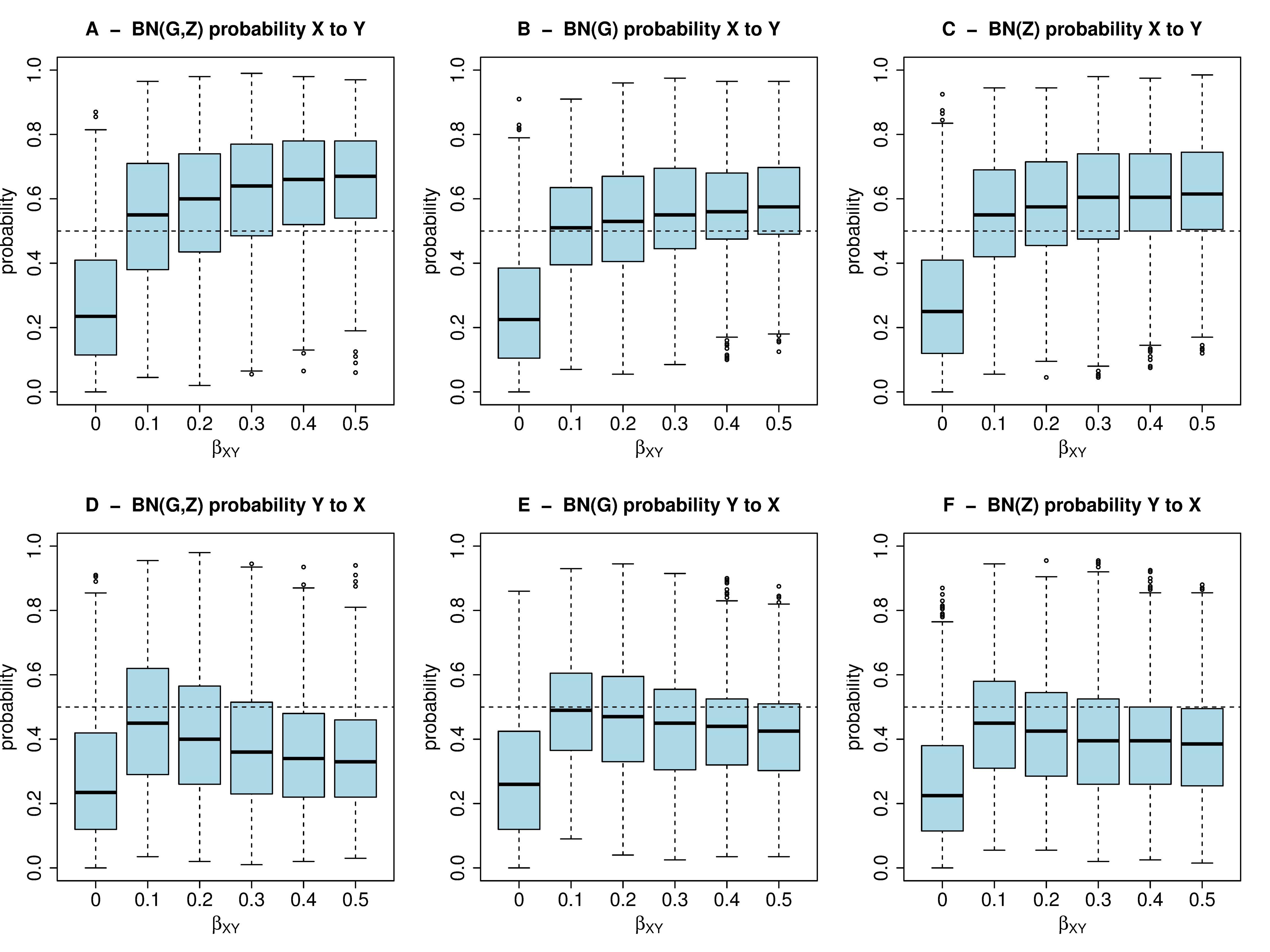

Supplement: S13 Fig — (TIF) [file pgen.1008198.s013.tif]

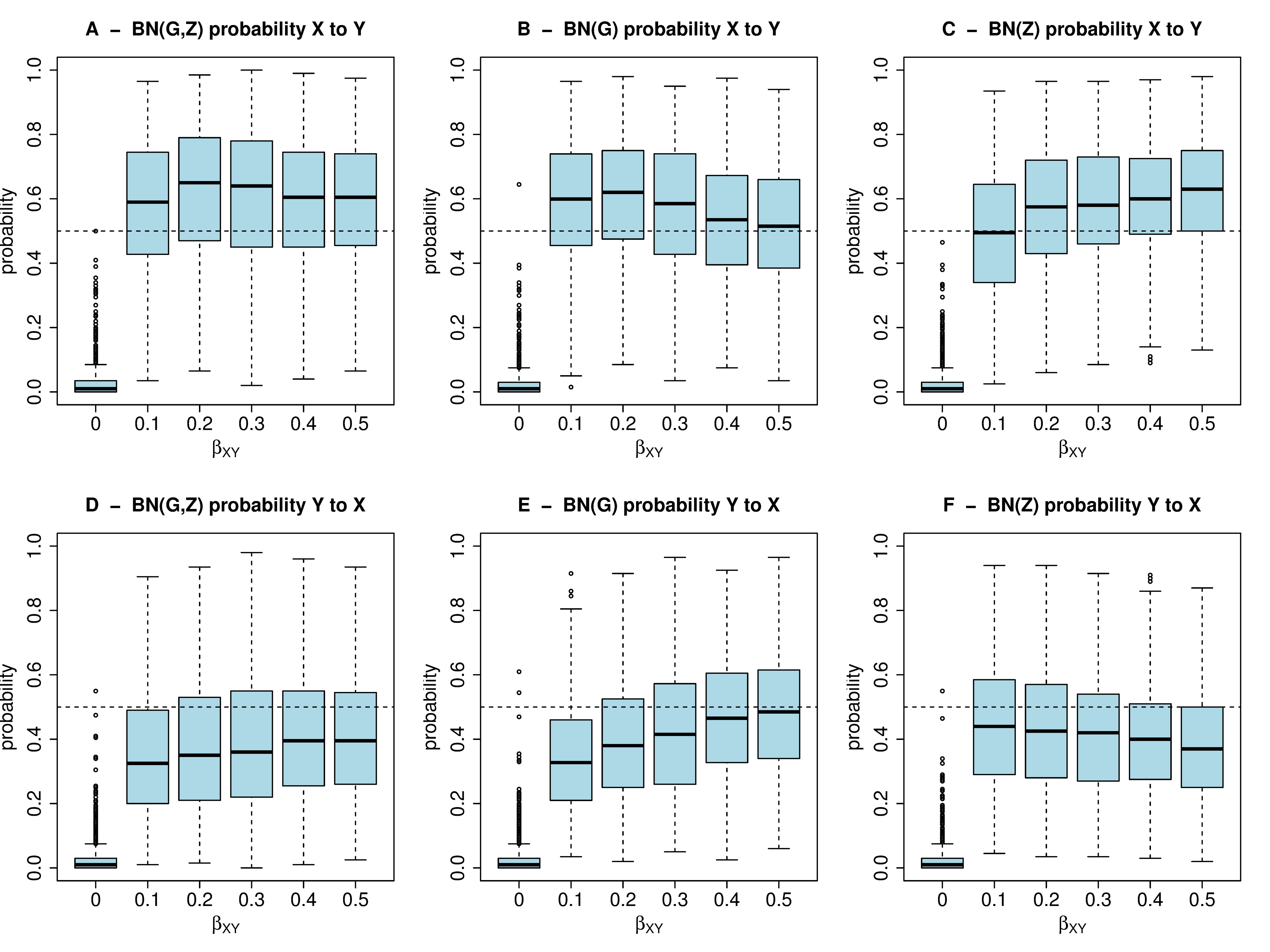

Supplement: S14 Fig — (TIF) [file pgen.1008198.s014.tif]

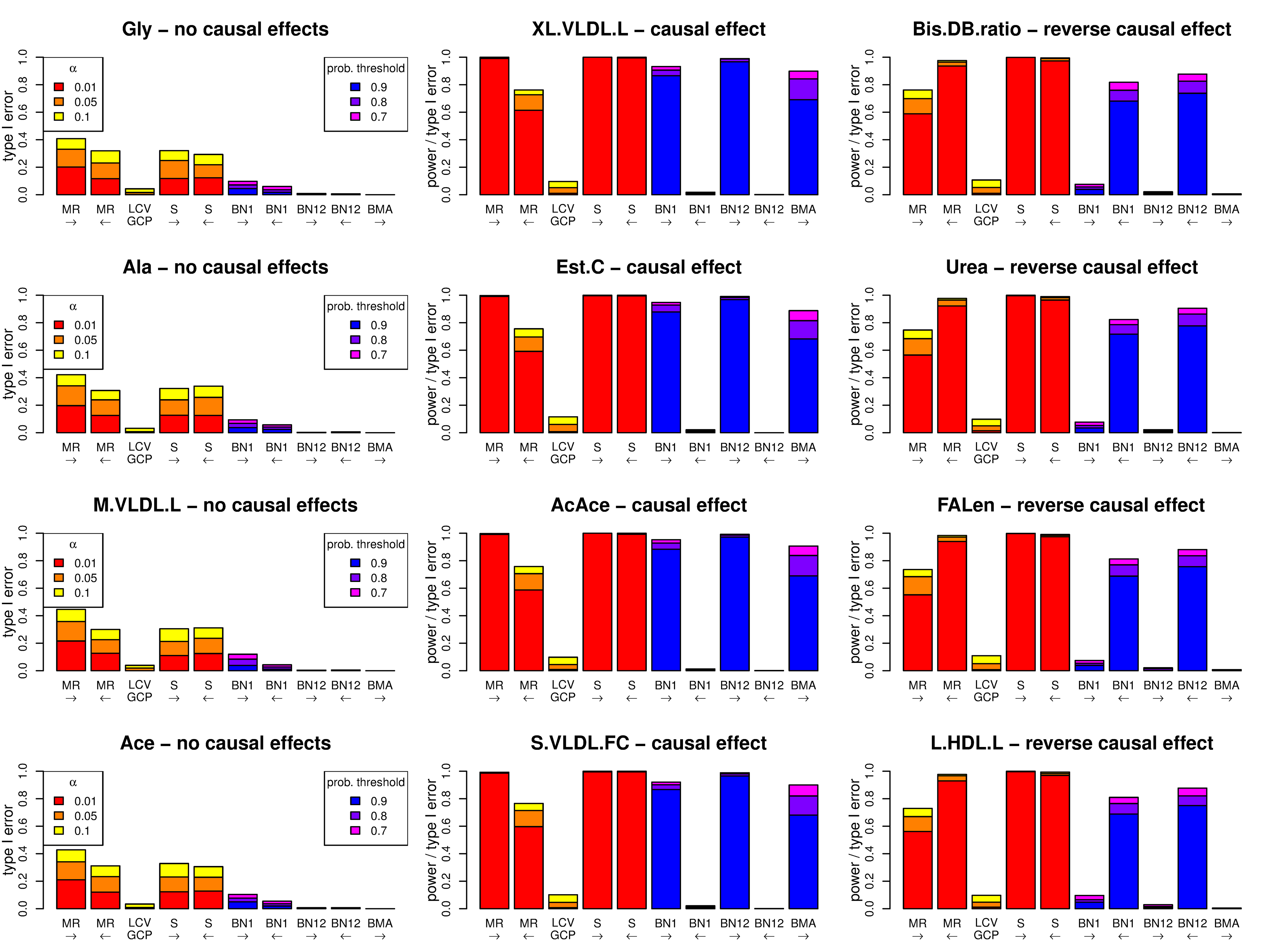

Supplement: S15 Fig — The model involves 12 metabolites, an outcome Y, 150 SNPs affecting the metabolites, 75 other SNPs affecting Y, and 9775 SNPs with no effect. Four metabolites (middle panels) have a causal effect on Y, four metabolites (right hand panels) have a reverse causal effect from Y to the metabolite, and four metabolites (left hand panels) have no effects to Y in any direction. The left-to-right arrows show tests for a causal effect from the metabolite to Y, and right-to-left arrows show tests from Y to one of the metabolites. MR: Mendelian randomization using an allele score as an instrumental variable for one of the metabolites or Y. LCV: latent causal variable methods where GCP denotes the genetic causality proportion test (testing the null hypothesis that GCP = 0), while LCV with left-to-right or right-to-left arrows corresponds to testing the null hypothesis that GPC = 1 (implying that the metabolite causes trait Y) or that GPC = −1 (implying that Y causes the metabolite), respectively. S: SMUT, using SNPs as random effect variables for one of the metabolites or Y. B1: Bayesian network consisting of one metabolite, Y and the two corresponding allele score variables. B12: Bayesian network consisting of all 12 metabolites, Y and all corresponding allele score variables. BMA: multivariable MR based on Bayesian model averaging (MR-BMA). (TIF) [file pgen.1008198.s015.tif]
